# Gray Matter and Regional Brain Activity Abnormalities in Subclinical Hypothyroidism

**DOI:** 10.3389/fendo.2021.582519

**Published:** 2021-02-24

**Authors:** Yang Zhang, Yaqiong Yang, Bo Tao, Qingguo Lv, Su Lui, Li He

**Affiliations:** ^1^ Neurology Department, West China Hospital of Sichuan University, Chengdu, China; ^2^ Huaxi MR Research Center, Department of Radiology, West China Hospital of Sichuan University, Chengdu, China; ^3^ Department of Endocrinology and Metabolism, West China Hospital of Sichuan University, Chengdu, China

**Keywords:** subclinical hypothyroidism, resting-state functional MRI, low-frequency fluctuation, levothyroxine, optimized voxel-based morphometry

## Abstract

**Background:**

Subclinical hypothyroidism (SCH) brain structure and resting state of functional activity have remained unexplored.

**Purpose:**

To investigate gray matter volume (GMV) and regional brain activity with the fractional amplitude of low-frequency fluctuations (fALFF) in subclinical hypothyroidism (SCH) patients before and after treatment.

**Material and Methods:**

We enrolled 54 SCH and 41 age-, sex-, and education-matched controls. GMV and fALFF of SCH were compared with controls and between pre- and post-treatment within SCH group. Correlations of GMV and fALFF in SCH with thyroid function status and mood scales were assessed by multiple linear regression analysis.

**Results:**

Compared to controls, GMV in SCH was significantly decreased in Orbital part of inferior frontal, superior frontal, pre-/postcentral, inferior occipital, and temporal pole gyrus. FALFF values in SCH were significantly increased in right angular, left middle frontal, and left superior frontal gyrus. After treatment, there were no significant changes in GMV and the local brain function compared to pre-treatment, however the GMV and fALFF of the defective brain areas were improved. Additionally, decreased values of fALFF in left middle frontal gyrus were correlated with increased mood scales.

**Conclusion:**

In this study we found that patients with SCH, the gray matter volume in some brain areas were significantly reduced, and regional brain activity was significantly increased. After treatment, the corresponding structural and functional deficiencies had a tendency for improvement. These changes may reveal the neurological mechanisms of mood disorder in SCH patients.

## Introduction

Subclinical hypothyroidism (SCH) is defined as serum thyroid-stimulating hormone (TSH) level elevated with normal levels of serum free thyroxine (1). Thyroid hormones play an important role in the development and maturation of the nervous system. It participates in the differentiation and migration of neurons, formation of myelin, establishment of synapses and formation of dendrites. It also affects the function of cholinergic and serotonin neurotransmitter in the central nervous system (2–4). Thyroid hormone receptors are widely expressed in the limbic system and the limbic system is involved in memory and mood regulation (5).

The current clinical findings demonstrated that effect of SCH on emotional status was controversial. Some studies suggested that the incidence of depressive symptoms in the SCH patients was significantly higher (6–9). However, some studies suggested that there was no correlation between thyroid hormone levels and cognitive impairment or mood disorders in patients with SCH (10–12). In addition, some studies found that after 3–6 months of levothyroxine replacement treatment SCH patients’ working memory function, intelligence scores and depression anxiety symptoms were significantly improved (13–18). However, some studies have found that replacement therapy did not completely relieve the symptoms of depression (19).

The radionuclide imaging and MRI studies have been used to investigate the neurological mechanisms of mood regulation of SCH from the aspects of glucose metabolism, brain blood flow and brain function. Krausz Y et al. found that SCH patients had a decrease in blood flow at the occipital lobe and posterior cingulated gyrus, and the abnormalities of blood perfusion were not reversed after the recovery of thyroid function. No correlation was found between blood flow and the depression scale and the mini-mental state examination (MMSE) scale (20, 21). In contrast, Bauer M et al. included patients with hypothyroidism and SCH, found that there were abnormalities in glucose metabolism in limbic system (mainly the amygdala, hippocampus, and cingulate gyrus) before the treatment, but can be completely reversed after treatment. The remission of depressive symptoms of SCH patients was negatively correlated with glucose metabolism in cingulate gyrus (22). In addition, the task state functional MRI (fMRI) study found the SCH patients’ damage in brain function (mainly cognitive function). Zhu found that the working memory-related network at frontal lobe was impaired by BOLD-fMRI, after about 6 months of thyroid hormone therapy patient’s memory and executive function recovered (23). However, there were no studies to explore the changes in brain structure and resting state of functional activity in SCH.

In this study, we followed SCH patients to estimate emotional changes before and after treatment. We also used the optimized VBM based on 3D-T1 magnetic resonance imaging to explore changes in the gray matter structure of patients with SCH (24, 25). The fractional amplitude of low-frequency fluctuations (fALFF) method was used to investigate the abnormalities of regional brain function activity in patients with SCH based on resting-state functional MRI (RS-fMRI) (26). And we further explore the relationship between abnormal brain regions and emotional sores changes in patients with SCH.

## Materials and Methods

### Subjects

This study was approved by the Ethics Committee of West China Hospital, Sichuan University. Informed consent was obtained from all participants prior to their inclusion in this study.

Patients diagnosed with SCH were recruited from the endocrinology department of West China Hospital. Subjects were excluded if they had psychiatric disorders, autoimmune diseases (such as low T3 syndrome, SLE, Sjogren’s syndrome, rheumatoid arthritis), had taken thyroid hormone or glucocorticoid in the last 2 months and other endocrine disorders. 41 age-, sex-, and education level-matched healthy controls enrolled. All healthy controls came from recruitment advertisement, which described the purpose and plan of this trial in detail. According to the requirements of the ethics committee, we also give some reimbursement to the healthy controls. All subjects were assessed with the Hamilton anxiety rating scale (HAMA) and Hamilton depression rating scale (HAMD) by a trained psychiatrist. Psychiatrist were blinded to healthy controls and patients. All subjects were right-handedness (determined by the Annett Hand Preference Questionnaire) (27).

### MRI Image Acquisition

All participants were scanned at West China Hospital of Sichuan University. The German SIEMENS MAGNETOM Skyra syngo 3.0T MR scanning system and 8-channel head coils were used to receive signals. The subject was supine on the scanning table, with head fixed by a foam pad, wearing earplugs to minimize noise, keeping eyes closing. The subjects were instructed to stay relaxed and not to think anything, but also to stay awake. The images were read by two professional radiologists, and subjects with any morphological abnormalities in the MR images were excluded. Radiologists who perform MRI scanning and images reading were blinded to healthy controls and patients.

A 3D spoiled gradient echo sequence was used to scan to obtain a high-resolution 3D brain structure (3D-T1 image). The scan parameters were as follows: TR/TE: 8.5 ms/3.4 ms, flip angle 12°, FOV: 240 mm × 240 mm, volume Element: 0.47 mm × 0.47 mm × 1.00 mm, matrix: 512 × 512, slice thickness 1 mm. A total of 156 axial plane images were collected in the whole brain. The resting state brain function image was obtained using a gradient echo-planar imaging sequence (EPI). The scanning parameters were as follows: TR/TE: 2000 ms/30.0 ms, flip angle 90°, FOV: 240 mm × 240 mm, voxel: 3.75 mm × 3.75 mm × 5.0 mm, matrix: 64 × 64, slice thickness 5 mm. A total of 30 axial plane structure maps and 200 functional image volumes were collected for each participant.

The first MRI acquisition of the SCH patients was performed within 1 week after the diagnosis of SCH without any medicine. The levothyroxine replacement therapy was started the next morning after the MRI. When the patient was treated for at least 3 months and the thyroid function had been returned to the normal range, the second MRI was performed. The control group had only one MRI acquisition and was also performed within 1 week after the detection of thyroid function. All subjects’ emotional scale assessments were performed on the day of the patient’s MRI scan.

### Image Processing and Analysis

3D-T1 images were processed using an optimized voxel-based morphometry method using SPM8 (Statistical Parametric Mapping, http://www.fil.ion.ucl.ac.uk) Software. Based on the Matlab (The Mathworks Inc, USA) platform, resting state data was processed using DPARSF (http://www.restfmri.net) and REST (http://www.restfmri.net). The fractional amplitude of low-frequency fluctuations (fALFF) is calculated as follows: The FFT (fast Fourier transform) is used to convert the time domain into the frequency domain to obtain the whole brain energy spectrum, and the power spectrum in the frequency range of 0.01–0.08 Hz is performed. After the square root is calculated, the ALFF value was obtained, and the total ALFF within this range is added to obtain the total value thereof, which is divided by the total amplitude value of the entire frequency band of 0.01–0.25 Hz, to obtain fALFF. Then, the fALFF value for each voxel was divided by the total brain mean fALFF value for normalization.

### Statistical Analysis

Two-sample t-tests and chi-square tests were conducted using SPSS 22.0 (SPSS Inc, Chicago, IL, USA) to compare the demographic data among the SCH patients and the healthy control group. we also use two-sample t-test to compare the data between SCH patients before and after treatment. A two tailed p value < 0.05 was set to be considered statistically significant. The results of continuous data were presented as mean (standard deviation). The differences of voxel-based GMV between SCH patients and HC were analyzed using Statistical Parametric Mapping (SPM8, http://www.fil.ion.ucl.ac.uk/spm). The significance of group differences was set at the threshold of p < 0.05 corrected by False Discovery Rate (FDR) correction.

## Results

A total of 54 patients with SCH and 41 normal controls were enrolled in this study, and only 26 patients completed the second MRI scan. The subjects were all right-handed. There was no statistically significant difference between the age distribution, sex ratio, and education degree between the patients and the healthy control group. The average TSH level of 54 patients was 7.54 ± 2.35 mU/L (4.78~15.87), which was higher than the normal range of 0.27–4.2 mU/L. FT4 level was 14.66 ± 1.83 (12.00~20.14) pmol/L, in the normal range of 12.0~22.0 pmol/L. The average TSH level of the control group was 2.04 ± 0.78 (0.35~4.09) mU/L, and the FT4 was 16.08 ± 2.15 (12.00~22.00) pmol/L, which were at the normal range. SCH TSH levels were significantly higher than those in the control group (P<0.001), and the level of FT4 was significantly lower than those in the control group (P=0.001) (**Table 1**).

**Table 1:** Baseline characteristics of SCH patients and healthy controls that completed the study.

	SCH before treatment (n = 54)	Healthy controls (n = 41)	T value	P value
Age	33.50 ± 8.76	32.75 ± 9.06		
Sex (female/male)	53/1	38/2		
Education (year)	14.31 ± 3.21	15.05 ± 3.91	24.987	0.001
TSH (mU/L)	7.54 ± 2.35*	2.04 ± 0.78	11.901	0.001
FT4 (pmol/L)	14.66 ± 1.83*	16.08 ± 2.15	48.370	<0.001
24-HAMD	5.76 ± 5.15*	3.05 ± 3.49	5.545	<0.001
14-HAMA	5.28 ± 4.23*	2.73 ± 2.78	8.092	0.001
	SCH before treatment (n=26)	SCH after treatment (n=26)		
TSH (mU/L)	7.65 ± 2.76**	2.35 ± 1.08***	11.054	0.001
FT4 (pmol/L)	14.95 ± 2.00**	17.23 ± 2.36***	37.257	<0.001
24-HAMD	5.31 ± 4.47**	3.46 ± 2.97***	6.050	0.001
14-HAMA	4.88 ± 3.84**	3.42 ± 2.63***	6.451	<0.001

“*”54 patients with SCH compared with Controls, P<0.01. “**”: 26 patients with SCH compared with Controls, P<0.01. “***”: SCH patients after treatment compared with SCHs before treatment, P<0.001. SCH, subclinical hypothyroidism; TSH, thyroid-stimulating hormone; FT4, free thyroxine. 24-HAMD: Hamilton depression rating scale. 14-HAMA: Hamilton anxiety rating scale.

In SCH group, the mean score of HAMD was 5.76 ± 5.15. The mean score of HAMA was 5.28 ± 4.23. The score of HAMD scale (P=0.001) and HAMA scale (P=0.001) were significantly higher than that in the control group (see **Table 1**).

After treatment of levothyroxine, only 26 patients were followed up regularly and completed the second MR image. The time interval between the two magnetic resonance image acquisitions was 150.6 ± 35.0 (91~242) days. After treatment, the score of HAMA and HAMD scale were significantly decreased (P=0.001) (See **Table 1**).

### Changes in Gray Matter Volume of Cerebral Cortex in Patients With SCH

**Figure 1:**
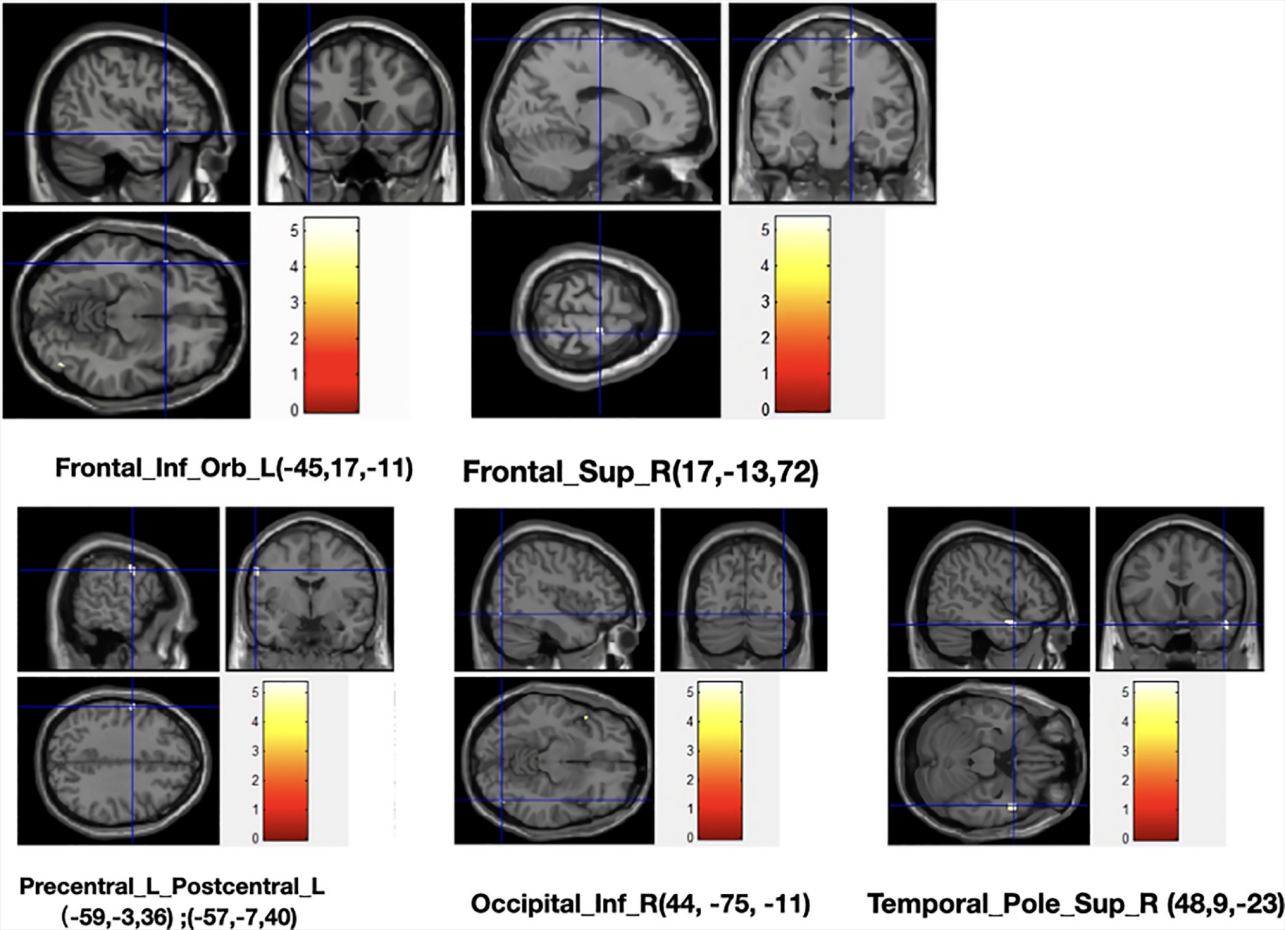
Voxels with significant GM decrease in SCH patients compared to healthy controls.

Compared with the control group with normal thyroid function, the volume of gray matter of the left frontal inferior orbital gyrus, the right frontal superior gyrus, the right inferior occipital gyrus, the left precentral and postcentral gyrus were significantly reduced (FDR corrected, see **Table 2**, **Figure 1**); After treatment, gray matter volume in the brain regions that had differences before treatment all showed a tendency to increase, however, we did not found statistical significant (FDR corrected).

**Table 2:** Gray matter volumes in SCH patients compared with healthy controls.

Anatomic regions	MNI coordinates (x, y, z)	Voxels	T value	*P*
GMV: SCH < healthy controls				
Frontal_Inf_Orb_L	−45,17, −11	10	4.64	0.034
Frontal_Sup_R	17, −13, 72	47	4.73	0.044
Pcecentral_L_Postcentral_L	−59, −3,36/−57, −7,43	66	4.79/4.20	0.032/0.037
Occipital_Inf_R	44, −75, −11	7	4.55	0.037
Temporal_Pole_Sup_R	48, 9, −23	130	5.37	0.032

Comparisons were performed at p < 0.05 corrected by False Discovery Rate (FDR) correction. The x, y, and z coordinates indicate the peak locations in the MNI space. A positive T value means increased GMV in SCH compared with controls. Abbreviations: MNI, Montreal Neurological Institute Coordinate System or Template; R, Right; L, Left.

**Figure 2:**
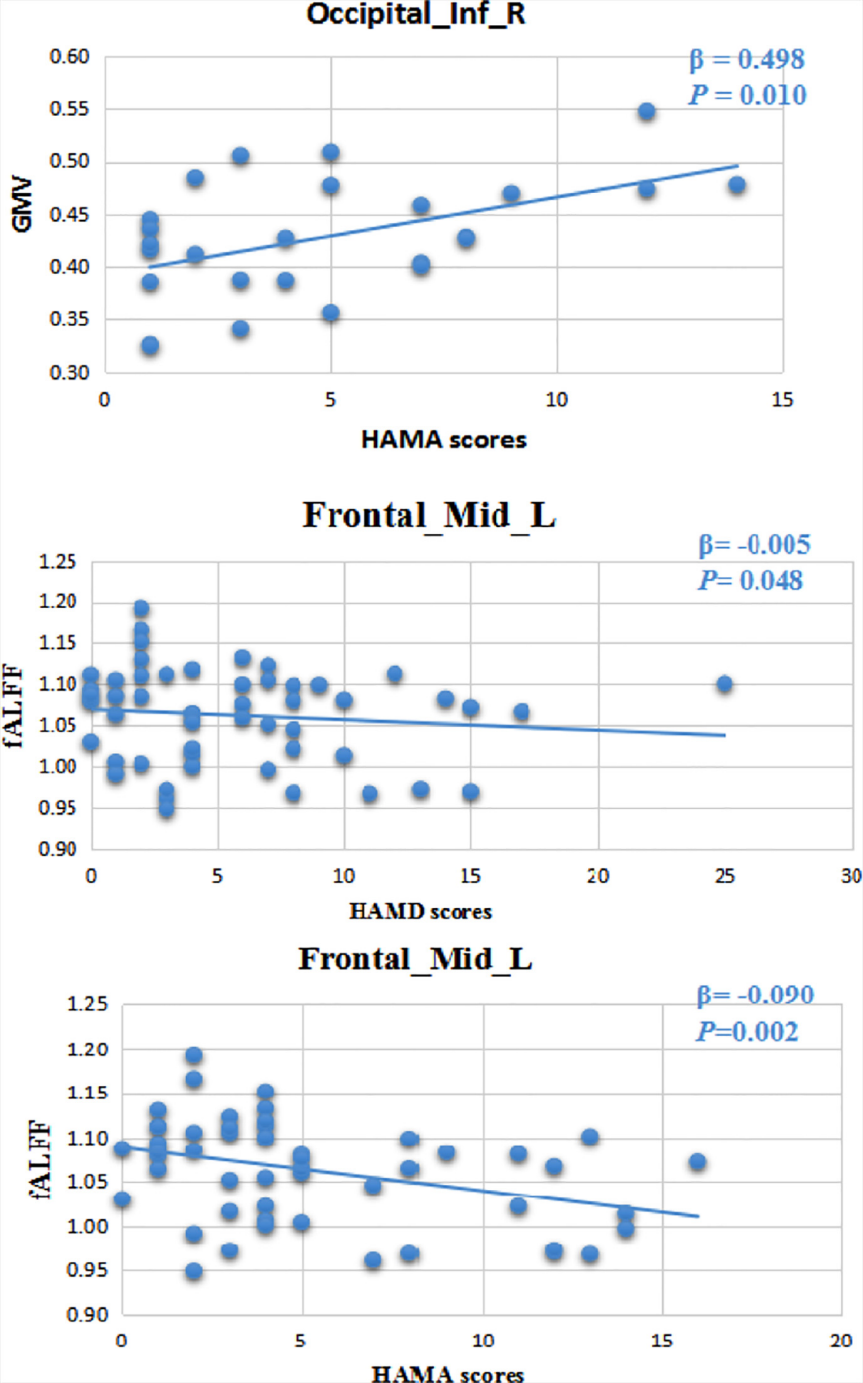
Volume of gray matter in the inferior occipital cortex had a correlation with the HAMA scale (β=0.498, P=0.010). There was a correlation between regional brain function activity (fALFF) in the left middle frontal gyrus and HAMA (β=-0.090, P=0.002) and HAMD (β=-0.005, P=0.048). 24-HAMD: Hamilton depression rating scale. 14-HAMA: Hamilton anxiety rating scale.

Multiple linear regression analysis showed that there was a correlation between the volume of gray matter in the inferior occipital cortex and the HAMA scale (β=0.498, P=0.010, **Figure 2**).

### Changes of Brain Activity in Patients With SCH

Compared with the control group the right angular gyrus, left frontal middle gyrus, and left frontal superior gyrus had significantly higher fALFF in patients with SCH (see **Table 3**). After treatment, the fALFF of these areas of brain had a tendency to decrease but no statistically difference was found (FDR Corrected).

**Table 3:** FALFF values changes in SCH patients compared with healthy controls.

Anatomic regions fALFF: SCH > healthy controls	MNI coordinates (x, y, z)	Voxels	T value	*P*
Angular_R	39, −57, 45	3	5.18	0.016
Frontal_Mid_L	−33, 18, 39	3	5.39	0.016
Frontal_Sup_L	−18, 18, 51	2	5.21	0.016

Comparisons were performed at p < 0.05 corrected by False Discovery Rate (FDR) correction. The x, y, and z coordinates indicate the peak locations in the MNI space. MNI, Montreal Neurological Institute Coordinate System or Template; R, Right; L, Left.

Multiple linear regression analysis showed that there was a correlation between regional brain function activity in the left middle frontal gyrus and HAMA (β=-0.090, P=0.002) and HAMD (β=-0.005, P=0.048) scale scores (**Figure 2**).

## Discussion

This study found that the volume of gray matter in the left frontal orbital gyrus, right superior frontal gyrus, right inferior occipital gyrus, the left precentral and postcentral gyrus, and right upper temporal pole was significantly reduced in patients with SCH and the fALFF of the right angular gyrus, the left frontal middle gyrus, left superior frontal gyrus increased significantly compared to healthy controls. Depression and anxiety scores of SCH patients were significantly higher than that of healthy controls.

We found there was a correlation between gray matter volume in the right inferior occipital gyrus and anxiety scores (HAMA). FALFF in the left middle frontal gyrus had a correlation with depression (HAMD) and anxiety (HAMA) scores respectively. After levothyroxine therapy, there were no significant changes of cortical gray volume and fALFF in the SCH group.

The main finding of our study was that the gray matter volume of SCH is significantly lower than that of the normal control group and these brain areas were mainly concentrated on the anterior part of the brain, mainly on the bilateral frontal lobes. We also found that the fALFF of the left frontal lobe was also significantly increased compared with the healthy control group. These findings were consistent with previous studies. Some studies suggested that the volume of gray matter in the frontal gyrus of patients with anxiety disorders was significantly reduced compared with the healthy control group (28, 29). Nagamachi S et al. confirmed that there was abnormal blood perfusion in prefrontal gyrus in hypothyroidism patients with severe depressive disorder (30). Shin YW Schraml FV’s study found high regional homogeneity and brain metabolic activity in the bilateral pre- and postcentral gyri of patients with hypothyroidism, and the high metabolic activity in these regions was associated with severe anxiety and depression in those patients (31, 32). Kumar et al. (33) also found that when compared to healthy controls, SCH patient showed significantly decreased in somato-motor network (SMN) and right fronto-parietal attention network (RAN) and increased in default mode network. The prefrontal cortex area is associated with executive function, attention and inhibition reactions. It participates in the non-spatial working memory tasks (34). The medial frontal cortex is also a part of the default network and responsible for body’s sense of smell, taste, vision, hearing and touch (including pleasant feelings or pain) (35). The lateral prefrontal cortex plays a regulatory role in this conversion process, while the medial prefrontal cortex participates in the decision-making process after value conversion (36). The anxiety and depression scores of patients with SCH were significantly higher than those of the healthy control group, which may be related to a decrease in frontal gray matter volume, and an increase in fALFF in the frontal gyrus may be a functional compensation of reduced volume. Prior to treatment of SCH patients, there was a correlation between emotional scores (HAMA and HAMD scales) with fALFF value in frontal lobe, this finding also may confirm that the role of this brain area in mood regulation.

After levothyroxine replacement therapy, we found that brain areas with statistically significant decreased in gray matter volume before treatment had an increased trend after treatment, and brain areas with significant differences in fALFF also showed a tendency to decrease after treatment. However, statistically significant was not achieved. This may suggest that after the treatment of SCH, the brain structure and function defects have a tendency to improve. These results showed the beneficial effect of treatment of SCH with RS-fMRI. Anna et al’s RS-fMRI study (37) also suggested that reduction in levothyroxine treatment in SCH induced deficits in working memory tasks.

This study also has some limitations. First, the study did not follow up the healthy control group at the same period. It was not clear whether the gray matter volume and regional brain function after treatment were completely recovered to normal. Second, we cannot guarantee that all subjects were not thinking about anything while performing resting-state fMRI image acquisition, we cannot rule out the possibility of bias. Third, although we used fALFF instead of ALFF to observe changes in the brain’s regional functional activity in patients with SCH, we cannot completely eliminate the impact of physiological noise (such as heartbeat, respiratory rate, etc.). Fourth, in order to exclude the influence of old age and comorbidities on neuropsychological function and imaging data, the study included young subjects and could not explain the effects of SCH on neuroanatomy and functional networks in the older population. Fifth, the patients and healthy people in this study were nearly all female; it maybe leads to bias. Therefore, the results of our study are mainly applicable to female SCH populations. Finally, although the sample size of patients in this study was more than previous studies (previous studies were mostly between 10 and 25), a larger sample size is still needed.

## Conclusion

In conclusion, we found that patients with SCH, the gray matter volume in some brain areas were significantly reduced, and regional brain activity was significantly increased. After treatment, the corresponding structural and functional deficiencies had a tendency for improvement. These changes may reveal the neurological mechanisms of mood disorder in SCH patients.

## Data Availability Statement

The raw data supporting the conclusions of this article will be made available by the authors, without undue reservation.

## Ethics Statement

The studies involving human participants were reviewed and approved by Ethics Committee of West China Hospital, Sichuan University. The patients/participants provided their written informed consent to participate in this study.

## Author Contributions

LH, YZ, QL, SL, and BT conceived of the presented idea. YY developed the theory and performed the computations. All authors contributed to the article and approved the submitted version.

## Funding

This work was supported by the National Key R&D Program of China and Project of Science (Funding Number: 2018YFC1311401), Technology Support Program of Sichuan Provincial Science and Technology Department (Funding Number: 2014SZ0005), and 1·3·5 project for disciplines of excellence–Clinical Research Incubation Project, West China Hospital, Sichuan University (Funding Number:2018HXFH022).

## Conflict of Interest

The authors declare that the research was conducted in the absence of any commercial or financial relationships that could be construed as a potential conflict of interest.
